# Reactive Oxygen Species-Sensitive Nanophotosensitizers Composed of Buthionine Sulfoximine-Conjugated Chitosan Oligosaccharide for Enhanced Photodynamic Treatment of Cancer Cells

**DOI:** 10.3390/ijms252312609

**Published:** 2024-11-24

**Authors:** Hyo Young Lee, Ji-Sun Park, Taeyu Grace Kim, Taeyeon Kim, Do Hoon Kim, Jejung Yun, Young-IL Jeong

**Affiliations:** 1 Department of Radiological Science, Dong-Eui University, Pusan 47340, Republic of Korea; lhy250@deu.ac.kr; 2 Interdisciplinary Program of Perfume and Cosmetic, Chonnam National University, Gwangju 61186, Republic of Korea; cnc3121@naver.com; 3 Wellesley College, 106 Central Street, Wellesley, MA 02481, USA; tk105@wellesley.edu; 4 College of Arts and Sciences, University of Pennsylvania, 20 Cohen Hall, 249 South 36th St, Philadelphia, PA 19104, USA; taeyeonk@sas.upenn.edu; 5 Tyros Biotechnology Inc., 75 Kneeland St. 14 Floors, Boston, MA 02111, USA; kimdh0495@hanmail.net; 6 Jeonnam Bio Foundation (JBF), Food Research Center, Naju City 58275, Jeonnam, Republic of Korea; jjyoung4@hanmail.net; 7 Department of Dental Materials, College of Dentistry, Chosun University, Gwangju 61452, Republic of Korea

**Keywords:** photodynamic therapy, chlorin e6, reactive oxygen species, buthionine sulfoximine, glutathione, chitosan

## Abstract

The efficacy of photodynamic therapy (PDT) based on traditional photosensitizers is generally limited by the cellular redox homeostasis system due to the reactive oxygen species (ROS) scavenging effect of glutathione (GSH). In this study, buthionine sulfoximine (BSO), a GSH inhibitor, was conjugated with the amine group of chitosan oligosaccharide (COS) using a thioketal linker (COSthBSO) to liberate BSO and chlorine e6 (Ce6) under oxidative stress, and then, Ce6-COSthBSO NP (Ce6-COSthBSO NP), fabricated by a dialysis procedure, showed an accelerated release rate of BSO and Ce6 by the addition of hydrogen peroxide, indicating that nanophotosensitizers have ROS sensitivity. In the in vitro cell culture study using HCT116 colon carcinoma cells, a combination of BSO and Ce6 efficiently suppressed the intracellular GSH and increased ROS production compared to the sole treatment of Ce6. In particular, Ce6-COSthBSO NP showed higher efficacy in the suppression of GSH levels and ROS production compared to the free Ce6 and Ce6/BSO combination. These results were due to the fact that Ce6-COSthBSO NP was efficiently delivered to the intracellular region, suppressed intracellular GSH levels, and elevated ROS levels. The in vivo animal tumor xenograft study demonstrated Ce6-COSthBSO NP being efficiently delivered to the tumor tissue, i.e., the fluorescence intensity in the tumor tissue was higher than those of other organs. The combination of Ce6 and BSO efficiently suppressed tumor growth compared to the sole treatment of Ce6, indicating that BSO might efficiently suppress GSH levels and increase ROS levels in the tumor microenvironment. Specifically, Ce6-COSthBSO NP showed the strongest performance in inhibition of tumor growth than those of Ce6 or the CE6/BSO combination, indicating that they were efficiently delivered to tumor tissue, increased ROS levels, and then efficiently inhibited tumor growth. We suggest that COSthBSO nanophotosensitizers are promising candidates for PDT treatment of cancer cells.

## 1. Introduction

Photodynamic therapy (PDT) is based on the photo-mediated treatment of human disease that involves specific wavelengths of light and photosensitizers [1]. PDT is composed of non-toxic components such as oxygen, light, and photosensitizers. Photosensitizers, which are chemical substances for the generation of molecular oxygen, are activated in conjunction with a specific wavelength of light and then produce reactive oxygen species to elicit cell death (phototoxicity) in the field of light irradiation, even though photosensitizers are freely dispersed in the human body after intravenous administration. That is, photosensitizers produce excessive amounts of reactive oxygen species (ROS) in the field of light irradiation and then kill abnormal cells or bacteria [1,2,3]. Due to these intrinsic features of PDT, it is regarded as a safe and efficient option for the diagnosis and treatment of cancer [1,3,4]. Principally, the therapeutic efficacy of PDT greatly depends on the photosensitizer. For example, 5-aminolevulic acid (5-ALA), which is a precursor of the porphyrin synthesis pathway, enhances the synthesis of porphyrin via the cellular heme biosynthetic pathway in cells [5]. 5-ALA has been used to visualize cancer and to resect by surgical treatment through fluorescence imaging [6,7]. 5-ALA-based PDT is regarded as an effective photosensitizer for gastrointestinal cancer [8,9]. Furthermore, chlorin e6 (Ce6), which is a second-generation photosensitizer, has strong absorption peaks at a wavelength of 664 nm [10]. Ce6 is known to exhibit higher ROS production than that of first-generation photosensitizers and has promising anticancer activity against various types of cancer [10,11,12,13,14]. Taber et al. reported that mono-L-aspartyl Ce6 (Npe6), a Ce6 derivative, showed efficient control of tumor regrowth (66%), even though the selectivity for destruction of the tumor was not apparent compared with normal skin at Npe6 [11]. Npe6 is known to show decreased skin photosensitivity due to its rapid clearance from the skin [12]. Furthermore, Ce6 and its derivatives are effective in treating gastrointestinal cancer and breast cancer [13,14].

However, some drawbacks of PDT still remain. For example, non-specific delivery, poor biodistribution, and rapid clearance from the circulation system of the human body of traditional photosensitizers can still cause light sensitivity and burning of the skin, even though they are not activated under the absence of light irradiation [15]. Furthermore, poor aqueous solubility of Ce6 is also problematic for clinical application. Since light has limitations in penetrating through the mucous layer of tissues or the surface of the skin, it is difficult to apply against tumors located deeper than 15 mm [16,17]. Various vehicles have been developed to solve these obstacles [4,18,19,20]. Xie et al. reviewed that nanocarriers are an ideal candidate for overcoming the drawbacks of traditional photosensitizers [18]. Nano-based vehicles that respond to light irradiation can be applied to deep-seated tumors and then promote tumor inhibition with reduced pain. Yu et al. reported that zeolitic imidazole framework 8 enhances cellular uptake, ROS generation, circulation time in vivo, and inhibition of tumor growth of Verteporfin [19]. In particular, nano-based vehicles can be designed to respond to the abnormal status of a tumor microenvironment since they can be delivered to tumor tissues by enhanced permeation and retention (EPR) effect [20].

The anticancer efficacy of photosensitizers is primarily influenced by the generation of ROS in the cancer cells [19,20]. Since the viability of cancer cells and/or diseased cells deeply depends on the intracellular ROS level, the efficacy of ROS production is considered the most important factor in PDT [20,21,22]. ROS production and the efficacy of accumulation in the diseased cells or tissues are critical factors when eradicating disease [21]. Even though photosensitizers under light irradiation produce an excessive amount of ROS, generated ROS in the field of light irradiation can be suppressed due to the defensive mechanism of the biological system [23]. In particular, L-glutathione (GSH), a typical ROS scavenger, contributes to controlling the intracellular levels of ROS to maintain biological homeostasis [24,25]. GSH contributes to the export of anticancer agents from cancer cells and is then involved in the induction of multi-drug resistance (MDR) [26]. Furthermore, a higher intracellular GSH level in cancer cells is known to be associated with resistance against hypericin-mediated PDT [27]. Theodossiou et al. reported that buthionine sulfoximine (BSO) significantly reduced the intracellular GSH level in MDA-MB 231 cells and then largely enhanced glutathione-S-transferase (GSTP1) sensitivity [27]. Furthermore, the depletion of GSH by BSO induces a complete reversal of MDR in lung carcinoma cells [26,27,28]. We also previously reported that BSO efficiently depleted the intracellular GSH level in HCT116 cells and then enhanced Ce6-based PDT [28]. Yoo et al. also reported that BSO reduced intracellular GSH levels and then synergistically killed tumor cells while applying PDT using Ce6-loaded nanoparticles [29].

In this study, we synthesized BSO-conjugated chitosan oligosaccharide (COS) using thioketal dicarboxylic acid (ThdCOOH) as a ROS-sensitive linker (COSthBSO) and fabricated Ce6-incorporated COSthBSO nanophotosensitizers (Ce6-COSthBSO NP) for efficient PDT application against HCT116 human colon cancer cells. We previously reported that HCT116 cells reveal relatively higher intracellular GSH levels than other kinds of cancer cells [28]. As far as we know, this is the first report on BSO-conjugated nanoparticles and the oxidative stress-responsive codelivery of BSO/Ce6 for application in PDT of colon cancer cells. We investigated the physicochemical properties, GSH depletion, and PDT efficacy of Ce6-incorporated COSthBSO nanoparticles against HCT116 cells in vitro.

## 2. Results

### 2.1. Synthesis and Characterization of COSthBSO Conjugates

**Figure 1:**
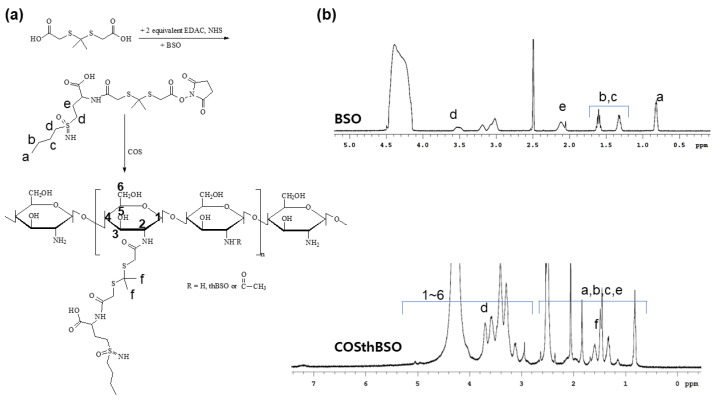
Synthesis scheme (**a**) and ^1^H NMR spectra (**b**) of COSthBSO conjugates.

To synthesize COSthBSO, the carboxyl group of ThdCOOH was activated with *N*-(3-Dimethylaminopropyl)-*N*′-ethylcarbodiimide hydrochloride (EDAC) and N-hydroxysuccinimide (NHS) to conjugate BSO, as shown in Figure 1a. Following this, another one was attached to the amine backbone of COS and then synthesized COSthBSO conjugates, as shown in Figure 1a. As shown in Figure 1b, ^1^H nuclear magnetic resonance (NMR) spectra showed that specific peaks of BSO were confirmed between 0.8 and 3.6 ppm. Protons of glucosamine were conformed at 3.0~5.4 ppm, while the methyl group of thioketal linker (ThdCOOH) and specific peaks of BSO were confirmed at 1.4 ppm and 0.8~2.6 ppm, respectively. These results indicated that BSO was properly grafted to the backbone of COS via a thioketal linker. The yield of conjugates was higher than 94.8% (*w*/*w*) by mass measurement. Based on UV absorption measurements, the experimental BSO content was 18.9% (*w*/*w*), while the theoretical content was 19.6 (Table S1 and Figure S1a). These results might be due to the fact that some of the BSO-ThdCOOH conjugates did not conjugate with COS and were then liberated during the dialysis procedure.

### 2.2. Preparation and Characterization of Ce6-Incorporated COSthBSO Nanoparticles

**Figure 2:**
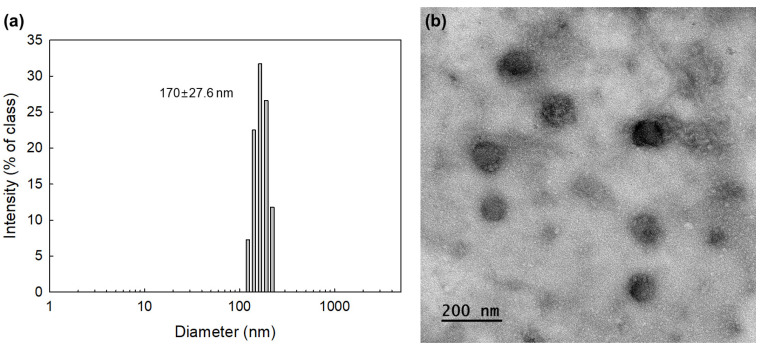
Particle size distribution (**a**) and transmission electron microscope image (**b**) of Ce6-incorporated COSthBSO nanophotosensitizers.

To fabricate the nanophotosensitizers, COSthBSO was dissolved in a DMSO/water mixture, and then Ce6 was added. This solution was dialyzed to form nanoparticles. Ce6-COSthBSO NP showed small diameters of less than 200 nm, as shown in Figure 2. The average particle size of the Ce6-incorporated COSthBSO nanoparticles was 170 ± 27.6 nm, as shown in Figure 2a and Table 1. Furthermore, they have spherical shapes with small diameters less than 200 nm, as shown in Figure 2b. The higher feeding ratio of Ce6 led to inducing higher drug contents and particle size, which also increased according to the increase in Ce6 contents as shown in Table 1.

**Figure 3:**
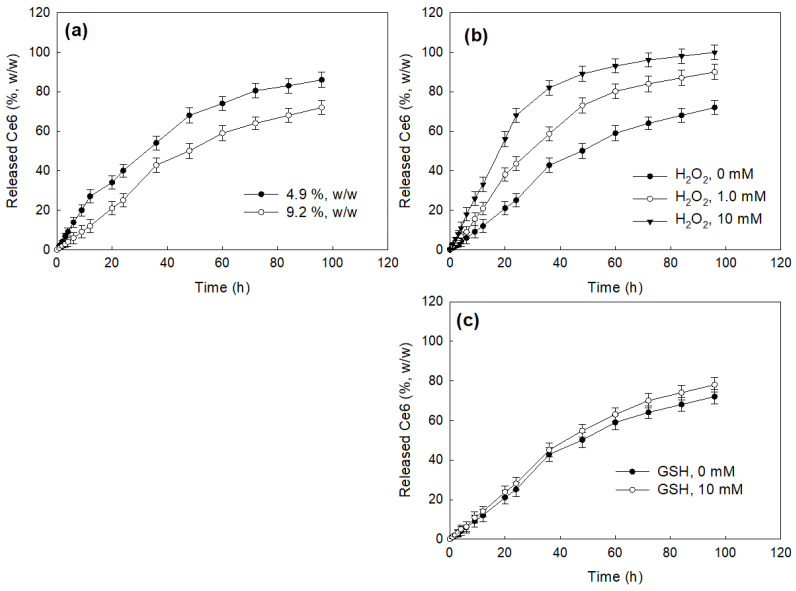
Ce6 release from Ce6-COSthBSO NP. The effect of Ce6 contents (**a**), H_2_O_2_ (**b**), and GSH (**c**).

To study Ce6’s release properties from the nanophotosensitizers, Ce6-COSthBSO NP were dispersed into phosphate-buffered saline (PBS), and then Ce6 release was performed in vitro, as shown in Figure 3. As shown in Figure 3a, Ce6 was continuously released from the nanophotosensitizers over 96 h. Since the thioketal group between COS and BSO has ROS sensitivity, H_2_O_2_ was added to the release media, as shown in Figure 3b. The Ce6 release rate accelerated according to the concentration of H_2_O_2_, indicating that COSthBSO nanophotosensitizers can be disrupted under oxidative stress and then release Ce6 faster than its normal status. GSH did not significantly affect the Ce6 release rate, as shown in Figure 3c. Furthermore, BSO release rate from COSthBSO nanoparticles was also accelerated according to the H_2_O_2_ concentration, while negligible amount of BSO was released in the absence of H_2_O_2_ (Figure S1b). These results indicate that COSthBSO nanoparticles have ROS sensitivity and liberate Ce6 under oxidative stress. Furthermore, the BSO release rate of COSthBSO nanoparticles was also accelerated by the addition of H_2_O_2_, indicating that COSthBSO nanoparticles themselves have ROS sensitivity and that they liberated BSO by oxidative stress.

### 2.3. In Vitro Cell Culture Study

**Figure 4:**
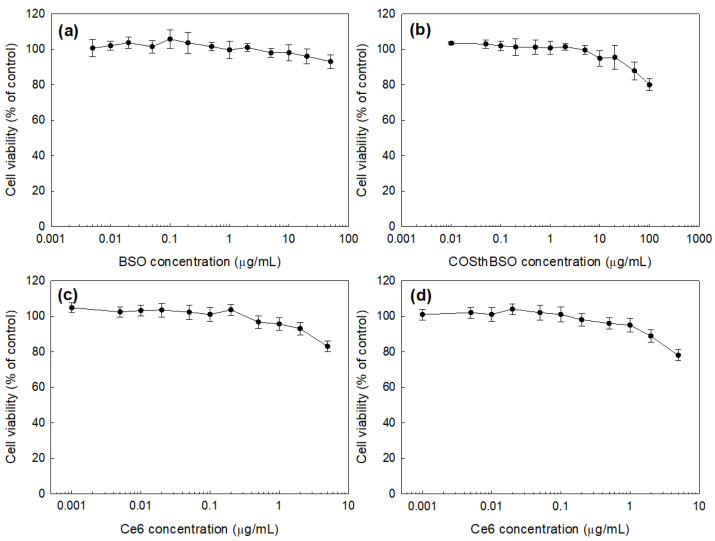
Intrinsic cytotoxicity of BSO (**a**), COSthBSO conjugates (**b**), Ce6 (**c**), and Ce6-COSthBSO NP (**d**). Ce6 contents of nanophotosensitizers was 9.2% (*w*/*w*).

Nanophotosensitizers, which have 9.2% (*w*/*w*) of Ce6 contents, were used for the cell culture study. Prior to studying the PDT effect of Ce6-COSthBSO NP, their intrinsic toxicities under dark conditions were studied using HCT116 human colon cancer cells, as shown in Figure 4. As shown in Figure 4a, BSO itself has no significant cytotoxicity against HCT116 cells until 50 µg/mL, i.e., cell viability was higher than 90% until 50 µg/mL. COSthBSO conjugates themselves also have low cytotoxicity against HCT116 cells, as shown in Figure 4b. Cell viability of the COSthBSO conjugate treatment was higher than 80% until 100 µg/mL. Otherwise, Ce6 itself and Ce6-COSthBSO NP have low cytotoxicity under dark conditions, as shown in Figure 4c,d. As shown in Figure 4c,d, cell viability was higher than 80% until 2 µg/mL Ce6 concentration of both Ce6 itself and Ce6-COSthBSO NP. As shown in Figure S2, the viability of normal cells, such as CCD986Sk human skin fibroblast cells (Figure S2a,b) and RAW264.7 mouse macrophage cells (Figure S2c,d), were higher than 90% until 50 µg/mL BSO. Their viabilities were also higher than 80% at 100 µg/mL COSthBSO conjugates. Ce6 or Ce6-COSthBSO NP did not significantly affect the viability of CCD986Sk cells and RAW264.7 cells, i.e., higher than 80% of the cells survived until 2 µg/mL Ce6 concentration, both for Ce6 itself and Ce6-COSthBSO NP. These results indicated that all of the components of Ce6-COSthBSO NP have low intrinsic cytotoxicity under dark conditions and did not significantly affect cell viability.

**Figure 5:**
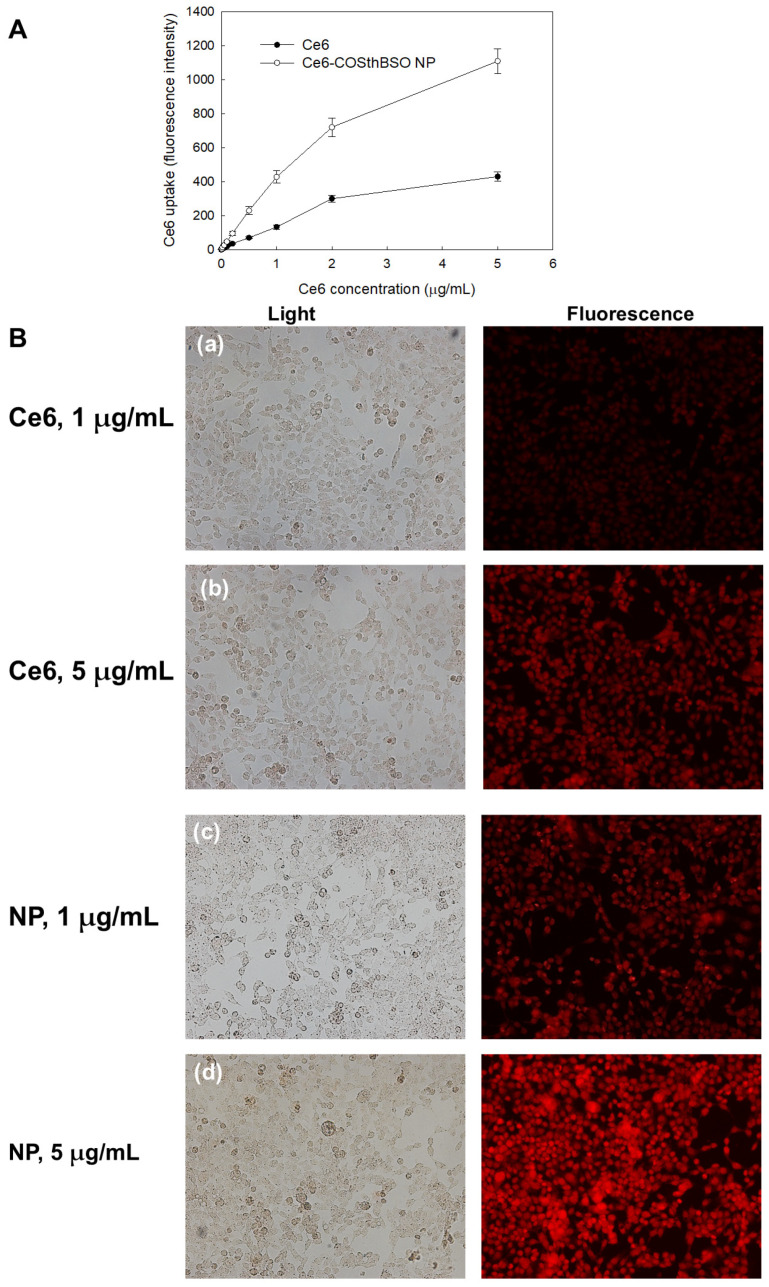
Ce6 uptake of Ce6 and Ce6-COSthBSO NP. (**A**) Ce6 uptake ratio. (**B**) Fluorescence microscopic images. (**a**) Ce6, 1 μg/mL; (**b**) Ce6, 5 μg/mL; (**c**) Ce6-COSthBSO NP (NP), 1 μg/mL as a Ce6 concentration; (**d**) Ce6-COSthBSO NP (NP), 5 μg/mL as a Ce6 concentration. Magnification 200×.

PDT’s efficacy of Ce6-COSthBSO NP was evaluated in vitro using HCT116 cells, as shown in Figure 5. Prior to evaluating PDT’s efficacy, the Ce6 uptake ratio was evaluated, as shown in Figure 5. As shown in Figure 5A, the Ce6 uptake ratio was increased dose-dependently by both Ce and Ce6-COSthBSO NP. In particular, the Ce uptake ratio of Ce6-COSthBSO NP was approximately three times higher than that of Ce6 itself, indicating that nanophotosensitizers are appropriate carriers for the delivery of Ce6 into intracellular regions. Fluorescence images of HCT116 cells supported these results, as shown in Figure 5B. The red fluorescence intensity of Ce6-COSthBSO NP was significantly higher than that of Ce6 itself, both for 1 µg/mL and 5 µg/mL.

**Figure 6:**
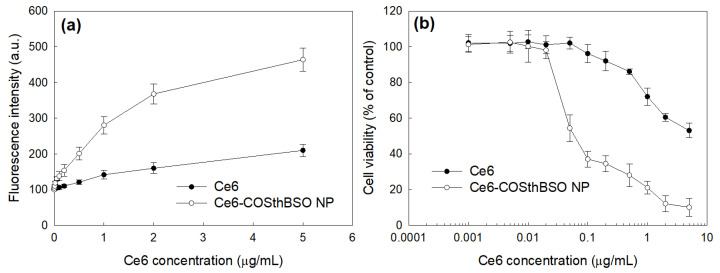
(**a**) ROS generation and (**b**) phototoxicity of Ce6 and Ce6-COSthBSO NP. For ROS generation and PDT treatment, cells were irradiated with an expanded homogeneous beam at 664 nm with a 2.0 J/cm^2^ light dose.

Figure 6 shows the ROS generation (Figure 6a) and cell viability (Figure 6b) after PDT. ROS generation of Ce6-COSthBSO NP was significantly higher than that of Ce6, as shown in Figure 6a. As expected, the phototoxicity of Ce6-COSthBSO NP was significantly higher than that of Ce6 itself, as shown in Figure 6b. These results indicated that Ce6-COSthBSO NP is a superior vehicle for PDT, i.e., the IC_50_ value of the nanophotosensitizers was significantly lower than Ce6, as shown in Table 2.

**Figure 7:**
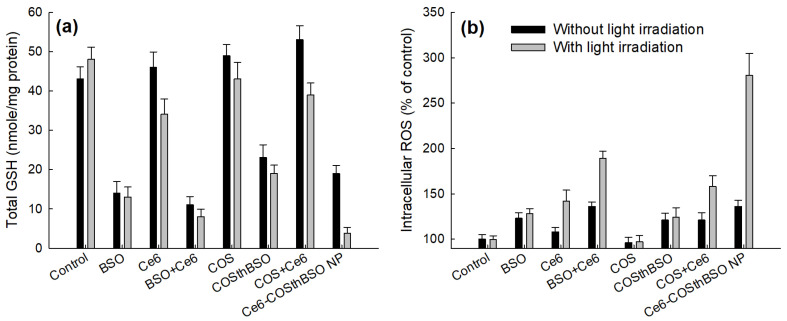
The effects of Ce6, BSO, COS, COSthBSO, and Ce6-COSthBSO NP (COSthBSO-Ce6 NP) on intracellular GSH contents (**a**) and ROS generation (**b**) in HCT116 cells with or without light irradiation. The light dose was 2.0 J/cm^2^ at 664 nm. Ce6 and BSO concentrations were 1 μg/mL and 2 μg/mL, respectively. COS and COSthBSO concentrations were 10 μg/mL and 10 μg/mL. For Ce6-COSthBSO NP (NP), Ce6 concentration was adjusted to 1 μg/mL. For control treatment, phosphate-buffered saline (PBS, pH 7.4, 0.01 M) was treated. Results are expressed as mean ± SD.

**Figure 8:**
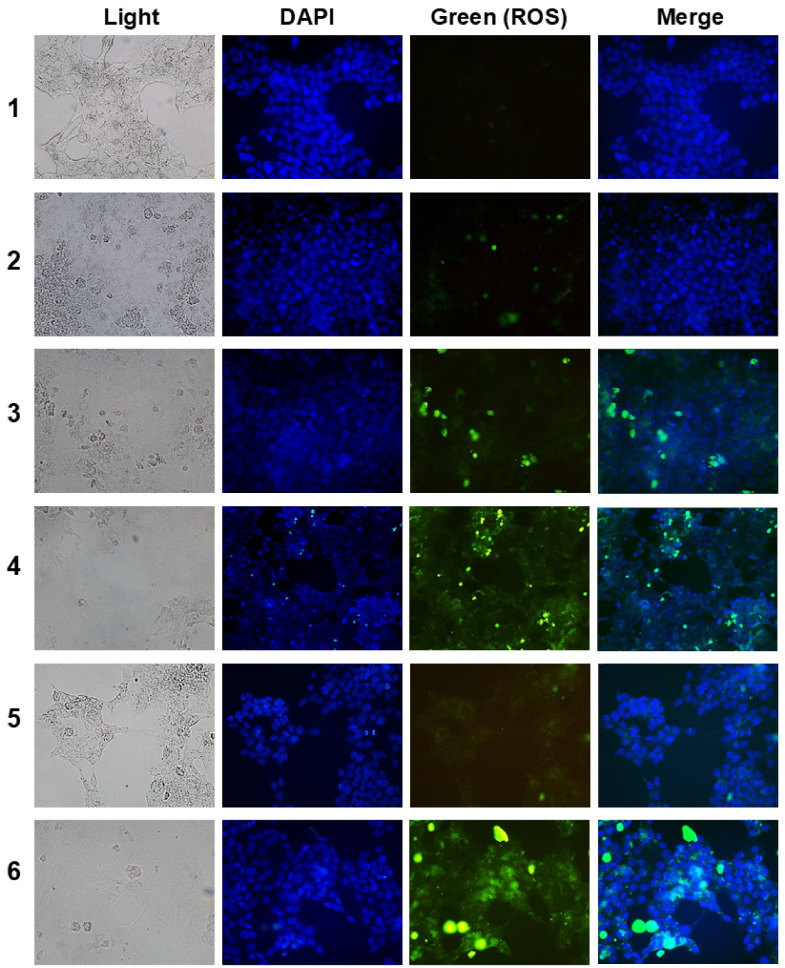
Fluorescence images of intracellular ROS in HCT116 cells. The light dose was 2.0 J/cm^2^ at 664 nm. Ce6 and BSO concentrations were 1 μg/mL and 2 μg/mL, respectively. COSthBSO concentrations were 10 μg/mL. For Ce6-COSthBSO NP, Ce6 concentration was adjusted to 1 μg/mL. For control treatment, phosphate-buffered saline (PBS, pH 7.4, 0.01 M) was treated. (**1**) Control; (**2**) free BSO; (**3**) free Ce6; (**4**) free Ce6 + free BSO; (**5**) COSthBSO nanoparticles; and (**6**) Ce6-incorporated COSthBSO nanophotosensitizers. Magnification, 400×.

Figure 7 shows the total intracellular GSH levels (Figure 7a) and their intracellular ROS levels (Figure 7b). As shown in Figure 7a, the BSO treatment significantly decreased the intracellular GSH levels in both the absence and presence of light irradiation. Particularly, a combination of BSO and Ce6 (BSO+Ce6) synergistically decreased the intracellular GSH levels rather than Ce6 itself or the COS+Ce6 combination. As expected, COSthBSO conjugates as empty nanoparticles also decreased the intracellular GSH levels. In particular, Ce6-incorporated COSthBSO nanophotosensitizers significantly decreased the intracellular GSH levels compared to other treatments. Practically, COS itself did not decrease the intracellular GSH levels, both in the absence and presence of light irradiation, while the combination of COS and Ce6 (COS+Ce6) also decreased the intracellular GSH levels. As expected, the combination of BSO and Ce6 significantly increased the intracellular ROS levels more than those of Ce6 itself or BSO alone. COSthBSO conjugates also increased the intracellular ROS levels, while COS did not affect the changes in intracellular ROS levels, while the combination of COS and Ce6 (COS+Ce6) also increased the intracellular GSH levels. Particularly, Ce6-COSthBSO NP significantly increased the intracellular ROS levels more than those of other treatments. These results indicated that Ce6-COSthBSO NP are superior to decreasing intracellular GSH levels and increasing intracellular ROS levels. As shown in Figure 8, the fluorescence intensity of Ce6-COSthBSO NP was significantly higher than that of other treatments. The combination of Ce6 and BSO also showed higher fluorescence intensity than those of Ce6 itself or BSO alone. These results indicated that Ce6-COSthBSO NP are suitable carriers for the generation of ROS in cancer cells.

### 2.4. In Vivo Animal Tumor Xenograft Study

**Figure 9:**
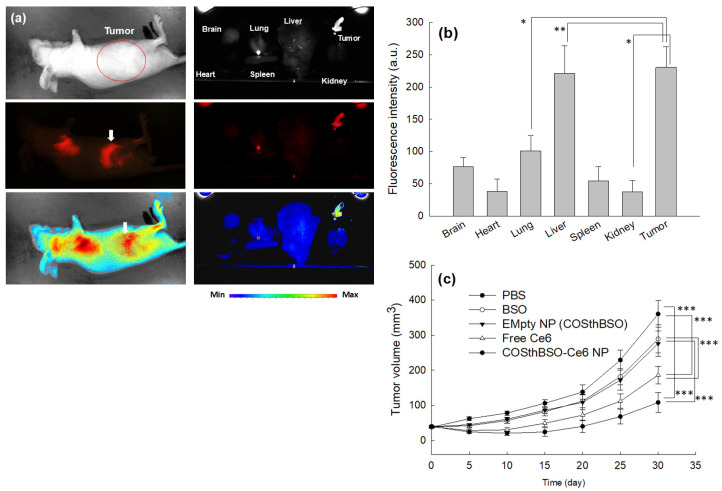
(**a**) Animal tumor imaging of HCT116-bearing tumor xenograft model. The BALb/C nude mouse was used. (**b**) Relative fluorescence intensity at each organ. Results are expressed as average ± SD of three mice. (**c**) The effects of PBS, BSO, COSthBSO conjugates (as empty nanoparticles), and free Ce6 or Ce6-incorporated COSthBSO nanophotosensitizers on the PDT of HCT116 tumor. (Ce6 dose = 10 mg/kg). *, ***: *p* < 0.01; **: *p* < 0.001.

The HCT116-bearing tumor xenograft model was used to investigate the PDT efficacy of Ce6-COSthBSO NP, as shown in Figure 9. Prior to investigating the PDT efficacy of nanophotosensitizers, the targeting capacity of Ce6-COSthBSO NP was studied using animal tumor imaging equipment, as shown in Figure 9a,b. The fluorescence intensity of the tumor mass was significantly higher than that of other organs. These results indicated that Ce6-COSthBSO NP are able to be delivered to tumors preferentially rather than other organs. As shown in Figure 9c, the tumor mass was time-dependently increased in all treatments. Practically, BSO alone or empty nanoparticles (COSthBSO) did not significantly affect the growth of the tumor volume. Otherwise, Ce6 or Ce6-COSthBSO NP efficiently inhibited the growth of the tumor volume by PDT on mice. In particular, Ce6-COSthBSO NP showed the highest efficacy in the inhibition of the growth of tumor volume. These results indicated that Ce6-COSthBSO NP are suitable vehicles for the inhibition of tumor growth.

## 3. Discussion

PDT’s efficacy of nanophotosensitizers is generally governed by ROS production in the intracellular compartment of cancer cells [17]. Even though ROS is present, both in normal cells and cancer cells, the ROS level is normally elevated in the cancer cells and tumor microenvironment [30]. Oxidative stress in the tumor microenvironment can be used as a biomarker or for drug-targeting issues [31,32,33,34]. For example, nanocarriers can be designed to be degraded at a high ROS level [31]. Pandya et al. reported that paclitaxel-loaded nanoparticles were degraded at 5 mM H_2_O_2_ and then showed significantly different size distributions, indicating that polymer nanoparticles responded to oxidative stress and then degraded [31]. Furthermore, their nanoparticles showed excellent anticancer activity compared to paclitaxel itself. ROS-sensitive nanoparticles based on β-cyclodextrin showed accelerated drug release at 1 mM H_2_O_2_ and then induced higher apoptosis of cancer cells [32]. Noddeland et al. also reported that the particle size of polymer nanoparticles was significantly increased, and then the drug release rate was accelerated in the presence of 10 mM H_2_O_2_ [33]. Our results also showed that the drug release rate of Ce6-COSthBSO NP was accelerated in the higher H_2_O_2_ concentration (Figure 3 and Figure S1b). ROS-sensitive nanophotosensitizers, having diselenide linkage, can be specifically delivered to the tumor tissues since the ROS level of the tumor microenvironment is elevated [34].

However, the antioxidant capacity also increases under oxidative stress to maintain homeostasis of physiological status in human tissues [35]. This status creates complex biological conditions in the tumor microenvironment. Redox homeostasis is an indispensable process in intracellular regions for maintaining the reducing and oxidizing reactions of cells [7]. That is, intracellular GSH levels, which are typical antioxidant molecules in the process of redox homeostasis, are also elevated when oxidative stress in cancer cells is elevated. GSH has a pivotal role in the protection of cells from attacks by foreign materials, oxidants, and mutagens [36]. These redox homeostasis phenomena in the intracellular region of cancer cells inhibit the anticancer activity of anticancer agents since the cytotoxicity of most anticancer agents is related to the generation of ROS [26,27,37]. Lee et al. reported that intracellular GSH levels of cancer cells significantly affect PDT’s efficacy, i.e., cancer cells having higher intracellular GSH levels are more resistant to PDT than those with lower intracellular GSH levels [23]. They also reported that inhibition of intracellular GSH levels induces more efficient PDT against cancer cells and then accelerates the apoptosis/necrosis of cells [28]. Intracellular GSH levels in cancer cells are also related to drug-resistance problems in cancer chemotherapy using traditional anticancer agents [38]. Cancer cells’ drug resistance is related to the intracellular GSH levels, which affect cell viability, i.e., higher intracellular GSH levels induce easier drug resistance against anticancer agents [37].

**Figure 10:**
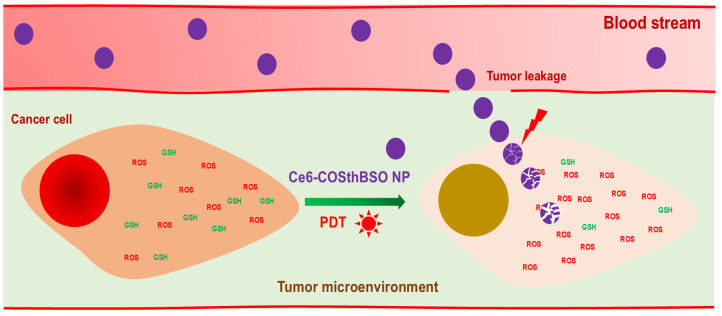
Schematic illustration of Ce6-incorporated COSthBSO nanophotosensitizers (Ce6-COSthBSO NP) against cancer cells.

One of the effective solutions for these problems is to suppress intracellular GSH secretion [28,36,38]. Actually, lowered GSH levels in cancer chemotherapy enhance the anticancer activity of traditional anticancer agents and inhibit drug-resistant phenomena [36]. Cui et al. reported that auranofin, a thioredoxin reductase, increased intracellular ROS levels with a depletion of intracellular GSH levels, and the addition of N-acetyl cysteine (NAC) prevented these reactions [39]. They also argued that BSO has a synergistic effect on the anticancer activity of auranofin through the depletion of GSH and elevation of ROS levels. In clinical studies, BSO effectively reduced the GSH contents in peripheral mononuclear cells of patients and enhanced the anticancer activity of traditional anticancer agents [40,41]. Then, the depletion of GSH in cancer cells was important for triggering the anticancer activity of anticancer agents [42]. Practically, BSO treatment with PDT has synergistic activity against cancer cells, which have higher intracellular GSH levels, and then it completely depletes the intracellular GSH levels at 1000 µM BSO [28]. However, BSO has a low or negligible synergistic effect against cancer cells having low intracellular GSH levels because cell viability was decreased according to the BSO concentration, and PDT’s efficacy was principally affected by the photosensitizer concentration [28]. In their report, HCT116 cells have the highest intracellular GSH level among various cancer cells [28]. In fact, BSO has negligible benefits in the depletion of GSH and elevation of intracellular ROS against cancer cells having the lowest intracellular GSH levels, such as SNU478 cells [28]. Even though BSO itself has low cytotoxicity against cells, BSO itself has contributed to the viability of cancer cells and normal cells, which have lower intracellular GSH levels; then it may be affected by the viability of normal cells. Practically, BSO decreases hepatic antioxidant levels, and it may affect the inflammatory reaction in vivo [43]. Depending on the types of tumor and organs, intra-tumoral GSH levels are quite different, and strategies based on a combination of BSO, Ce6, and/or BSO-conjugated nanocarriers are practically limited to the specific types of cancers having high GSH levels [44]. In our results, BSO addition to Ce6-based PDT against HCT116 cells resulted in synergistic anticancer activity in vitro. It effectively decreased the intracellular GSH levels and then contributed to producing ROS in HCT116 cells, as shown in Figure 7a. Furthermore, it contributed to the production of intracellular ROS in HCT116 cells. In particular, Ce6-COSthBSO NP significantly suppressed the intracellular GSH levels and enhanced the intracellular ROS levels compared to Ce6 itself and BSO alone (Figure 7). These results were due to the fact that the intracellular delivery of nanophotosensitizers was approximately three times higher than that of Ce6 itself (Figure 5a). These phenomena must be affected by the production of intracellular ROS with the suppression of intracellular GSH levels that then synergistically inhibit cell viability (Figure 6 and Figure 7). Even though BSO has low side effects in the human body, it also depletes the GSH levels in normal cells or tissues with an increase in oxidative stress, and then it needs to be concentrated in the region of disease [42,43]. In particular, COSthBSO nanophotosensitizers were preferentially concentrated in the tumor region, as shown in Figure 9a. These contribute to the improved anticancer activity of Ce6-COSthBSO NP in the in vivo HCT116-bearing tumor model (Figure 9c). Since the biodistribution of Ce6-COSthBSO NP was also at a high level in the liver, their PDT efficacies are limited to the specific tumors having high GSH levels, i.e., the PDT’s efficacy and safety may be affected by the adverse effects of BSO or COSthBSO conjugates [45]. Furthermore, their PDT efficacy should be defined according to the intracellular GSH levels in the in vivo tumor xenograft model. We will report the Ce6-COSthBSO NP using two types of cancer cells that have low and high intracellular GSH levels in the in vivo tumor xenograft model in the near future. Otherwise, Ce6 should also be concentrated in the tumor region due to its adverse effects that can be derived from non-specific delivery in the human body [15]. Ce6-COSthBSO NP has ROS-sensitivity and then effectively inhibited the growth of the tumor compared to Ce6 itself as illustrated in Figure 10. Principally, nano-scale particles are regarded as an ideal vehicle for the delivery of anticancer agents [46,47]. They can be delivered to the tumor site through enhanced permeation and retention (EPR) effects and then minimize the unwanted side effects of anticancer drugs against normal counterparts [42]. Ce6-COSthBSO NP can efficiently deliver Ce6 to the tumor site with minimal distribution to other organs, and then this might contribute to enhanced antitumor activity (Figure 9a,b). Chitosan nanoparticles are known to have acceptable biocompatibility in the biological system for clinical application, and BSO itself is already used in clinical studies to suppress GSH levels [48,49]. The biocompatibility of COSthBSO nanoparticles and Ce6-COSthBSO NP will be checked in a future report. At this moment, sole treatment of BSO or COSthBSO nanoparticles did not affect the survivability of mice, and all mice survived until 30 days.

## 4. Materials and Methods

### 4.1. Chemicals

Chitosan oligosaccharide (COS) was purchased from Tokyo Chemical Industry (TCI) Co., LTD. (Tokyo, Japan). Ce6 and Thioketal dicarboxylic acid (ThdCOOH) were obtained from Frontier Sci. Co. (Logan, UT, USA) and RuixiBiotech Co. Ltd. (Xi’an, China), respectively. L-Buthionine sulfoximine (BSO), N-(3-dimethylaminopropyl)-N’-ethylcarbodiimide hydrochloride (EDAC), N-hydroxysuccinimide (NHS), triethylamine (TEA), 2′,7′-dichlorofluorescin diacetate (DCFH-DA), 3-(4,5-dimethyl2-thiazolyl)-2, 5-diphenyl-2H-tetrazolium bromide (MTT), bicinchoninic acid (BCA) protein assay kit, glutathione reduced ethyl ester (GSH-OEt), dimethylsulfoxide (DMSO), phosphotungstic acid, and 2,2,2-tribromoethanol (avertin) were purchased from Sigma Aldrich Chem. Co. (St. Louis, MO, USA). Dialysis membranes, having molecular weight cut-off sizes (MWCO) of 1000 and 2000 g/mol, were purchased from Spectra/Por^TM^ Membranes (Rancho Dominguez, CA, USA). Cell culture media, such as Roswell Park Memorial Institute 1640 (RPMI 1640), fetal bovine serum (FBS), and antibiotics, were purchased from Life Tech. Inc. (Grand Island, NY, USA). Organic solvents and chemicals were used as extra-pure grade.

### 4.2. Synthesis BSO-Conjugated COS Copolymer Using Thioketal Linker (COSthBSO)

The COSthBSO conjugates were synthesized as follows: Primarily, ThdCOOH (224 mg) was dissolved in 5 mL of DMSO with 2 equivalent mol of EDAC (384 mg) and NHS (230 mg). This solution was magnetically stirred for 3 h to activate the carboxylic acid of ThdCOOH to make NHS-activated ThdCOOH. After that, one equivalent mole of BSO (222.3 mg) dissolved in 5 mL of DMSO/H_2_O (3/2, *v*/*v*) was added to this solution and then stirred for more than 6 h to synthesize the BSO-ThdCOOH conjugates. To this solution, COS (720 mg) dissolved in 5 mL of the DMSO/H_2_O mixture (4/1, *v*/*v*) was added and then further stirred for 24 h. Following this, unreacted chemicals, byproducts, and organic solvent was removed by dialysis procedure. The reactants were introduced into the dialysis membrane (molecular weight cut-off (MWCO): 1000 g/mol) and then dialyzed against deionized water for more than 2 days. The water was exchanged every 3 h intervals. Following this, the resultant solution was lyophilized to obtain the COSthBSO copolymer. The yield of the final products was calculated as follows: Yield (%, *w*/*w*) = [(weight of ThdCOOH + weight of BSO)/weight of COSthBSO conjugates] × 100. The yield was approximately 94.8% (*w*/*w*).

### 4.3. Characterization of Synthesized Products

The synthesized products were confirmed with 500 mHz NB Fourier transform (FT)-NMR spectroscopy (Varian Unity Inova; Varian Inc., Santa Clara, CA, USA). The BSO and/orCOSthBSO were dissolved in the DMSO/D_2_O mixtures, and then the ^1^H NMR spectra were analyzed.

### 4.4. Fabrication of Ce6-Incorporated Nanophotosensitizers

To fabricate the Ce6-incorporated nanoparticles (COSthBSO-Ce6 nanoparticles), 47.5 mg or 45 mg of COSthBSO reconstituted in 2 mL of deionized water was mixed with 5 mL of DMSO. Ce6 (2.5 mg or 5 mg) was dissolved in 2 mL of DMSO (2 mL) and added to the COSthBSO solution. This solution was sonicated with a bar-type sonicator for 30 s and then magnetically stirred for 10 min. This solution was introduced into a dialysis tube and then dialyzed against deionized water for 1 d to remove the unloaded Ce6 or organic solvent. To prevent saturation, the deionized water was exchanged at 3 h intervals for 1 day. Following this, the dialyzed solution was used to analyze or lyophilize for 2 days.

To measure the drug content, 5 mg of Ce6-incorporated nanoparticles were distributed in 3 mL of deionized water with sonication for 30 s and after that, 7 mL of DMSO was added to this solution, followed by 10 times dilution with DMSO. An UV–vis spectrophotometer (Genesys 10s UV-VIS spectrophotometer, Thermo Fisher Scientific, Waltham, MA, USA) was employed to measure Ce6’s concentration in the nanoparticles at 664 nm. COSthBSO conjugates (4.5 mg) dissolved in 10 mL of DMSO/H_2_O (7/3, (*v*/*v*), 10 mL) were diluted with DMSO 10 times, and then this solution was used to compensate for fluorescence intensity. Ce6 content (*w*/*w*, %) = (Ce6 weight/nanoparticle weight) × 100. Loading efficiency (*w*/*w*, %) = (Ce6 weight in the nanoparticles/Ce6 feeding weight) × 100.

### 4.5. Characterization of Nanophotosensitizers

The morphology of the nanoparticles was observed with transmission electron microscopy (TEM) (H7600, Hitachi Instruments Ltd., Tokyo, Japan). Twenty μL of the nanoparticle solution, fabricated as above, was placed onto a carbon film-coated grid and then dried at room temperature for more than 3 h. The nanoparticles were negatively stained with phosphotungstic acid (0.1%, *w*/*w* in H_2_O). TEM was operated at 80 kV for observation of the nanoparticles.

The particle size distribution of the Ce6-incorporated COSthBSO nanoparticles was measured with a Zetasizer (Nano-ZS, Malvern, Worcestershire, UK). To test redox sensitivity, H_2_O_2_ was added to phosphate-buffered saline (PBS, pH 7.4, 0.01 M), and then the nanoparticles were distributed into this solution with sonication for 30 s, followed by incubation at 37 °C for 4 h.

### 4.6. Drug Release Study

To study Ce6’s release from the nanoparticles, COSthBSO-Ce6 nanoparticles (5 mg) were distributed into 5 mL of PBS (0.01 M, pH 7.4) with 30 s sonication, and then this solution was introduced into a dialysis membrane (MWCO, 2000 g/mol). This was introduced into 45 mL of PBS (pH 7.4, 0.01 M) with or without H_2_O_2_. A drug release study was performed under shaking at 100 rpm and 37 °C. PBS was collected at predetermined time intervals to measure the liberated Ce6 concentration from the dialysis membrane. The collected PBS was discarded and replaced with fresh PBS. Ce6’s concentration in the PBS was measured with a UV–vis spectrophotometer at 664 nm. All experiments were expressed as the average ± standard deviation (SD) from three experiments.

### 4.7. Cell Culture Study

HCT116 human colorectal carcinoma cells were purchased from Korean Cell Line Bank, Co. (Seoul, Republic of Korea). The HCT116 cells were maintained in RPMI1640 (Gibco, Grand Island, NY, USA) medium supplemented with 10% heat-inactivated fetal bovine serum (FBS) (Invitrogen, Waltham, MA, USA) and 1% penicillin/streptomycin. Cells were cultured in a 5% CO_2_ incubator at 37 °C.

The intrinsic cytotoxicity of BSO, Ce6, and COSthBSO-Ce6 nanoparticles against HCT116 cells was as follows: HCT116 cells (2 × 10^4^ cells/well) seeded into 96-well plates were cultured overnight. Various concentrations of BSO, Ce6, COSthBSO conjugates, and COSthBSO-Ce6 nanoparticles were prepared with 100 µL of serum-free RPMI1640 medium. Media in the cell cultures were discarded, and the cells were washed with PBS and then treated with each chemical. The cells were cultured under dark conditions for 24 h. Following this, the cells were washed with PBS twice and the viability of the cells was evaluated with the MTT assay. MTT reagent (30 µL, 2 mg/mL in PBS) was added to the cells, followed by further incubation for 3 h. Media were discarded, and then 100 µL of lysis buffer solution (10% sodium dodecyl sulfate in 0.01 N HCl) was added to the cell culture to lyse the cells overnight. The absorbance of this solution was recorded at 570 nm with a microplate reader (Infinite M200 pro microplate reader, Tecan Trading AG, Mannerdorf, Switzerland).

### 4.8. Total GSH Level of HCT116 Cells

A total GSH detection kit (Enzo Life Sci. Inc., Farmingdale, NY 11735, USA) was used to measure the total intracellular GSH level of the HCT116 cells according to the manufacturer’s instructions. The protein concentration was calculated by measuring it with a BCA protein assay kit at 562 nm using an Infinite M200 pro microplate reader.

### 4.9. PDT Study In Vitro

HCT116 cells (2 × 10^4^ cells) in a 96-well plate were cultured overnight. After that, the cells were washed with PBS twice, and then various concentrations of BSO, Ce6, COSthBSO conjugates, and COSthBSO nanoparticles in 100 µL of serum-free medium were applied to cells for 2 h. After that, the media were discarded, the cells were washed with PBS twice, and then 100 µL of fresh medium was added. For PDT treatment, the cells were irradiated with an expanded homogeneous beam (SH Systems, Gwangju, Republic of Korea) at 664 nm. The light dose was 2.0 J/cm^2^. After that, the cells were incubated at 5% CO_2_ in an incubator (37 °C). Then, 24 h later, the viability of the cells was evaluated with the MTT assay at 570 nm using an Infinite M200 pro microplate reader.

Dark toxicity was carried out as follows: cells (2 × 10^4^ cells) seeded in a 96-well plate were treated with BSO, Ce6, COSthBSO conjugates, and COSthBSO nanoparticles in 100 µL of serum-free medium for 2 h. Following this, the medium was replaced with PBS twice to wash the cells; fresh medium (100 µL) containing 10% FBS was added, and then the cells were further incubated for 24 h. The viability of the cells was evaluated with the MTT assay at 570 nm using an Infinite M200 pro microplate reader.

### 4.10. Ce6 Uptake In Vitro

HCT116 cells (2 × 10^4^ cells/well) seeded into 96-well plates were cultured overnight at 5% CO_2_ in an incubator (37 °C). These cells were washed with PBS twice, and then the Ce6 or COSthBSO-Ce6 nanoparticles in serum-free medium were applied to cells, followed by further incubation at 37 °C for 2 h. The cells were washed with PBS twice, and then 50 μL of lysis buffer (GenDEPOT, Barker, TX, USA) was added to lyse the cells. The relative Ce6 uptake ratio was estimated under fluorescence conditions (excitation wavelength, 407 nm; emission wavelength, 664 nm) using an Infnite M200 pro microplate reader.

### 4.11. ROS Measurement In Vitro

For the measurement of intracellular ROS generation, the DCFH-DA method was used. BSO, Ce6, COSthBSO conjugates, and/or COSthBSO-Ce6 nanoparticles were applied to cells, and then the cells were irradiated, similar to the PDT study. Following this, media were discarded, and the cells were washed with PBS. DCFH-DA (final concentration: 20 μM) was diluted with the phenol red-free RPMI1640 medium and then further incubated for 2 h at 5% CO_2_ in an incubator (37 °C). After that, the media were discarded, and then the cells were washed with PBS twice. The phenol red-free RPMI1640 medium (100 μL) was added to the cells and then irradiated at 664 nm. The intracellular ROS level was evaluated by fluorescence (excitation wavelength, 485 nm; emission wavelength, 535 nm) using an Infinite M200 pro microplate reader.

### 4.12. PDT Study In Vivo Using HCT116-Tumor Bearing Mouse

To study PDT’s efficacy in vivo, a HCT116-tumor-bearing mouse was used. Further, 1 × 10^6^ HCT116 cells were subcutaneously injected into the back of nude BAL b/C mice (male, 20 g, five weeks old). When the diameter of the solid tumor in the backs of the mice reached larger than 4 mm, the mice were separated into four groups: (1) the PBS treatment group for the control; (2) the BSO treatment group as a positive control; (3) COSthBSO conjugates for the treatment group as a positive control; (4) Ce6 treatment group; and (5) the COSthBSO-Ce6 nanophotosensitizer treatment group. Each animal group was composed of 5 mice, and the injection volume of each chemical was 100 µL or 200 µL. The Ce6 dose was adjusted to 10 mg/kg. For Ce6 treatment, Ce6 dissolved in a Cremophor EL^®^/ethanol (1/1) mixture was diluted more than ten times with PBS (0.01 M, pH 7.4). Nanoparticles of the COSthBSO conjugates as empty nanoparticles or Ce6-COSthBSO NP (COSthBSO NP) fabricated as described above were sterilized with 1.2 µm syringe filters. These were intravenously (i.v.) administered through the tail vein of a mouse. After 2 days, the mice were anesthetized, and then PDT treatment (10 J/cm^2^, 664 nm) was carried out under dark conditions. To prevent the interference of light, the mice were covered with fabric material, except for the tumor mass. This day was recorded as day 0, and then light irradiation was performed once more three days later. At 5-day intervals, the diameter of the tumor was measured with vernier calipers, and then the tumor volume was calculated with the following equation: Tumor volume (mm^3^) = (length × width^2^)/2.

Fluorescence imaging of the mice was carried out as follows: HCT116-tumor bearing mice were used for observation of fluorescently. An aqueous solution of Ce6-COSthBSO NP (COSthBSO NP) was sterilized with 1.2 µm syringe filters. When the diameter of the solid tumor became larger than 7 mm, nanoparticles were i.v. administered via the tail vein. The injection volume was 200 µL. One day later, a mouse was sacrificed and then observed with a MaestroTM^2^ small animal imaging instrument (Cambridge Research & Instrumentation, Inc., Hopkinton, MA, USA).

Animal experiments were carried out under the guidelines of the Pusan National University Institutional Animal Care and Use Committee (PNUIACUC). The protocol for the animal study was reviewed and monitored by the PNUIACUC in accordance with their ethical procedures and scientific care and was approved (approval number: PNU-2017-1610).

### 4.13. Statistical Analysis

Statistical analysis of the results was evaluated with a Student’s *t*-test using the SigmaPlot^®^ program (Version 11.0, CA, USA). The *p*-value < 0.05 was determined as a minimal level of significance.

## 5. Conclusions

COSthBSO was synthesized through the conjugation of BSO using a thioketal linker. The thioketal linker was linked between the amine group of COS and BSO to endow ROS sensitivity. BSO was attached to the backbone of COS to suppress the intracellular GSH levels in cancer cells and to emphasize PDT’s efficacy of Ce6. BSO was successfully conjugated to the backbone of COS. COSthBSO nanoparticles and Ce6-COSthBSO NP have small particle sizes of less than 200 nm and are spherical shapes. Their particle sizes were increased according to the increase in Ce6 content. Ce6 was continuously released from Ce6-incorporated COSthBSO nanophotosensitizers over 4 days. Their Ce6 release rate was accelerated by the addition of hydrogen peroxide, indicating that nanophotosensitizers have ROS sensitivity, and then the Ce6 release rate can be accelerated in the presence of oxidative stress. BSO, COSthBSO nanoparticles, Ce6, and Ce6-COSthBSO NP have little or negligible cytotoxicity against HCT116 cells in the absence of light irradiation. Under light irradiation, Ce6-COSthBSO NP showed a significantly higher Ce6 uptake ratio, ROS generation, and cytotoxicity against HCT116 cells. Specifically, BSO synergistically decreased the total intracellular GSH levels. Furthermore, COSthBSO nanoparticles and Ce6-COSthBSO NP also decreased the total intracellular GSH levels as well as BSO alone. The intracellular ROS was also synergistically produced by the addition of BSO under Ce6-based PDT both of BSO plus Ce6 and Ce6-COSthBSO NP treatment. Specifically, intracellular ROS generation of Ce6-COSthBSO NP was significantly higher than that of other treatments. These results indicated that Ce6-COSthBSO NP have a superior capacity to suppress intracellular GSH levels and ROS production. In the in vivo animal tumor xenograft study, Ce6-COSthBSO NP were efficiently delivered to the tumor tissue, i.e., the fluorescence intensity in the tumor tissue was higher than that of other organs, and they efficiently inhibited the growth of the tumor mass. We suggest that COSthBSO nanophotosensitizers are promising candidates for the PDT treatment of cancer cells.

## Figures and Tables

**Table 1:** Characterization of Ce6-COSthBSO NP.

COSthBSO/Ce6 (mg/mg)	Drug Contents (%, *w*/*w*)		Particle Size ^1^ (nm)
	Theoretical	Experimental	
50/0	–	–	121 ± 20.8
47.5/2.5	5.0	4.9	170 ± 27.6
45/5.0	10.0	9.2	223 ± 32.7

^1^ Particle size distribution was based on intensity.

**Table 2:** Characterization of Ce6-COSthBSO NP.

	IC_50_ (µg/mL) ^1^
Ce6	1.68
COSthBSO-Ce6 NP ^2^	0.06

^1^ IC_50_ value was evaluated from Figure 5c. ^2^ COSthBSO-Ce6 NP: Ce6-COSthBSO NP.

## Data Availability

Data are contained within the article and Supplementary Materials.

## Supplementary Materials

The following supporting information can be downloaded at: https://www.mdpi.com/article/10.3390/ijms252312609/s1.
