# Supplementary material for: Reactive Oxygen Species-Sensitive Nanophotosensitizers Composed of Buthionine Sulfoximine-Conjugated Chitosan Oligosaccharide for Enhanced Photodynamic Treatment of Cancer Cells
Source: Int J Mol Sci. 2024 Nov 24;25(23):12609. doi: 10.3390/ijms252312609 (PMC11641636; doi:10.3390/ijms252312609)
Supplement: Supplementary file 1 [file ijms-25-12609-s001.zip › ijms-3290582-supplementary.pdf]

Supplementary materials

Experimental

Ultra-violet spectrophotometric analysis of BSO

To measure BSO content, COSthBSO (10 mg) were distributed in 10 ml PBS (pH 7.4, 0.01 M) with sonication for 30 s and, after that, hydrogen peroxide was added (final H_2O_2 concentration: 20 mM). This was incubated at 37°C (100 rpm) with shaker incubator for 2 days. Following this, resulting solution was diluted with PBS ten times and absorbance was measured with an UV-VIS spectrophotometer (Genesys 10s UV-VIS spectrophotometer, Thermo Fisher Scientific, Waltham, MA, USA) at 203 nm. To compensate UV absorption, similar concentration of COS, ThdCOOH and hydrogen peroxide was used as a blank.

BSO content (w/w, %) = (BSO weight/nanoparticle weight) × 100.

For BSO release study, COSthBSO (5 mg) were distributed in 5 ml PBS (pH 7.4, 0.01 M) with sonication for 30 s and then this solution was introduced into dialysis membrane (MWCO, 2000 g/mol). This was introduced into introduced into 45 mL PBS (pH 7.4, 0.01 M) with or without H_2O_2. PBS was collected at predetermined time intervals to measure the liberated BSO concentration from dialysis membrane. The collected PBS was discarded and replaced with fresh PBS. BSO concentration in the PBS was measured with UV-VIS spectrophotometer at 203 nm. All experiments were expressed as the average ± standard deviation (S.D.) from three experiments. similar concentration of COS, ThdCOOH and hydrogen peroxide was used as a blank.

Cell culture

Normal cells such as RAW264.7 mouse macrophage cells or CCD986sk human skin fibroblast cells was obtained from Korean Cell Line Bank (Seoul, Korea). RAW264.7 cells and CCD986sk cells were cultured in DMEM (Gibco, Grand Island, NY, USA) and IMDM (Gibco, Grand Island, NY, USA) medium, respectively. Cells were cultured in 37 C in a 5% CO_2 incubator and sub-cultured at intervals of 2-3 days. Media were supplemented with 10% heat-inactivated fetal bovine serum (FBS) (Invitrogen) and 1% penicillin/streptomycin.

In vitro cell cytotoxicity

Intrinsic cytotoxicity of BSO, Ce6 and COSthBSO-Ce6 nanoparticles against HCT116 cells were as follows: RAW264.7 cells and CCD986sk cells (2×10^4 cells/well) seeded into 96-well plates were cultured overnight. Various concentrations of BSO, Ce6, COSthBSO conjugates and COSthBSO-Ce6 nanoparticles were prepared with 100 µl serum-free media. Media in cell culture were discarded, washed cells with PBS and then treated with each chemical. Cells were cultured in the dark condition for 24 h. Following this, cells were washed with PBS twice and the viability of cells was evaluated with the MTT assay. MTT reagent (30 µl, 2 mg/ml in PBS) was added to cells following with further incubation for 3 h. Media were discarded and then 100 µl of lysis buffer solution (10% sodium dodecyl sulfate in 0.01 N HCl) was added to cell culture to lyse cells overnight. Absorbance of this solution was recorded at 570 nm with microplate reader (Infinite M200 pro microplate reader, Tecan Trading AG, Mannerdorf, Switzerland).

Table S1. BSO content in the COSthBSO nanoparticles

COSthBSO nanoparticles	Drug contents (%, w/w)		Particle size [2] (nm)
	Theoretical [1]	Experimental [1]	
	19.7	18.9	121±20.8

[1] Theoretical value of BSO was calculated from the theoretical M.W. of COSthBSO conjugates and experimental BSO contents was determined by UV absorption measurement.
[2] Particle size was similar to Table 1.

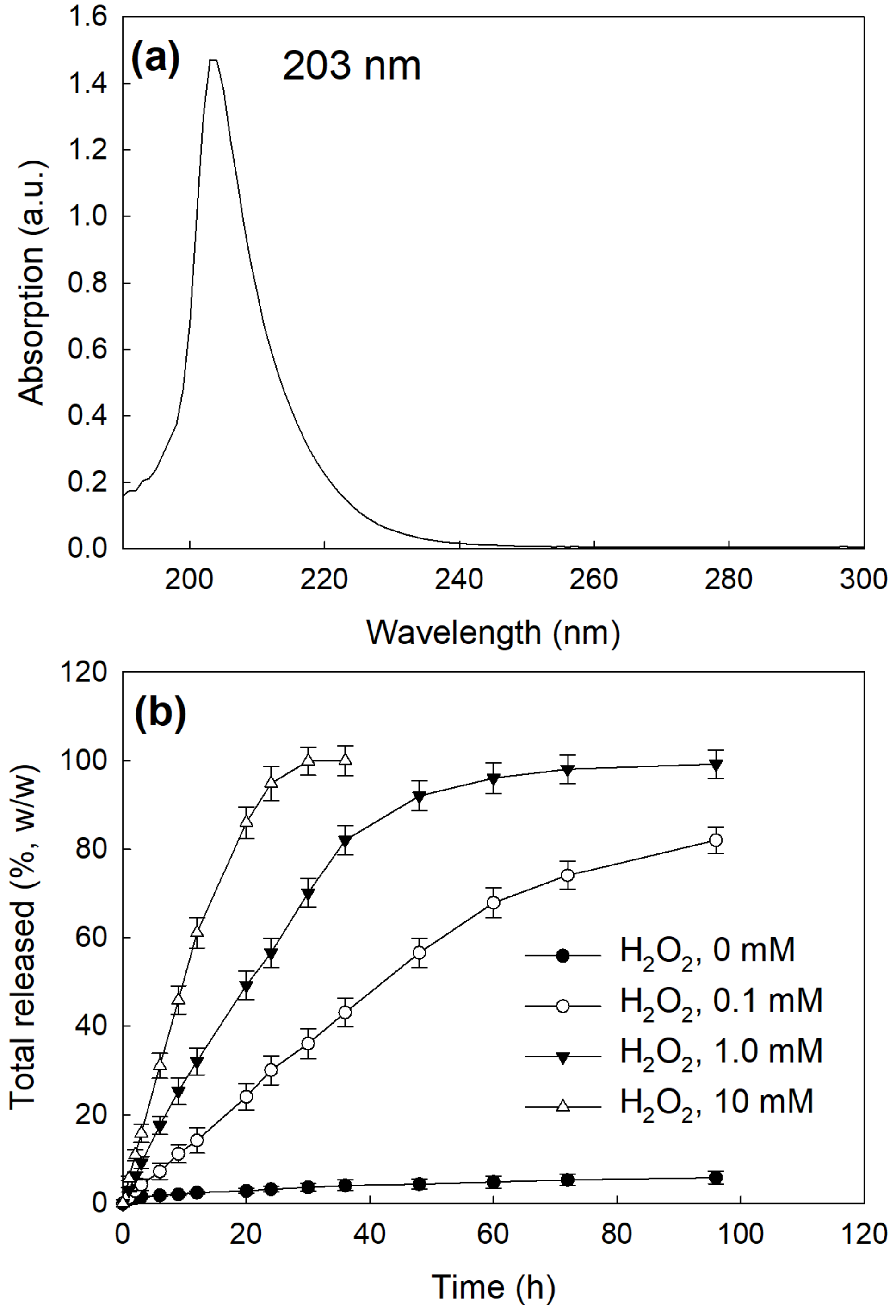

Figure S1 (a) UV absorption spectra of BSO. (b) BSO release from COSthBSO nanoparticles.

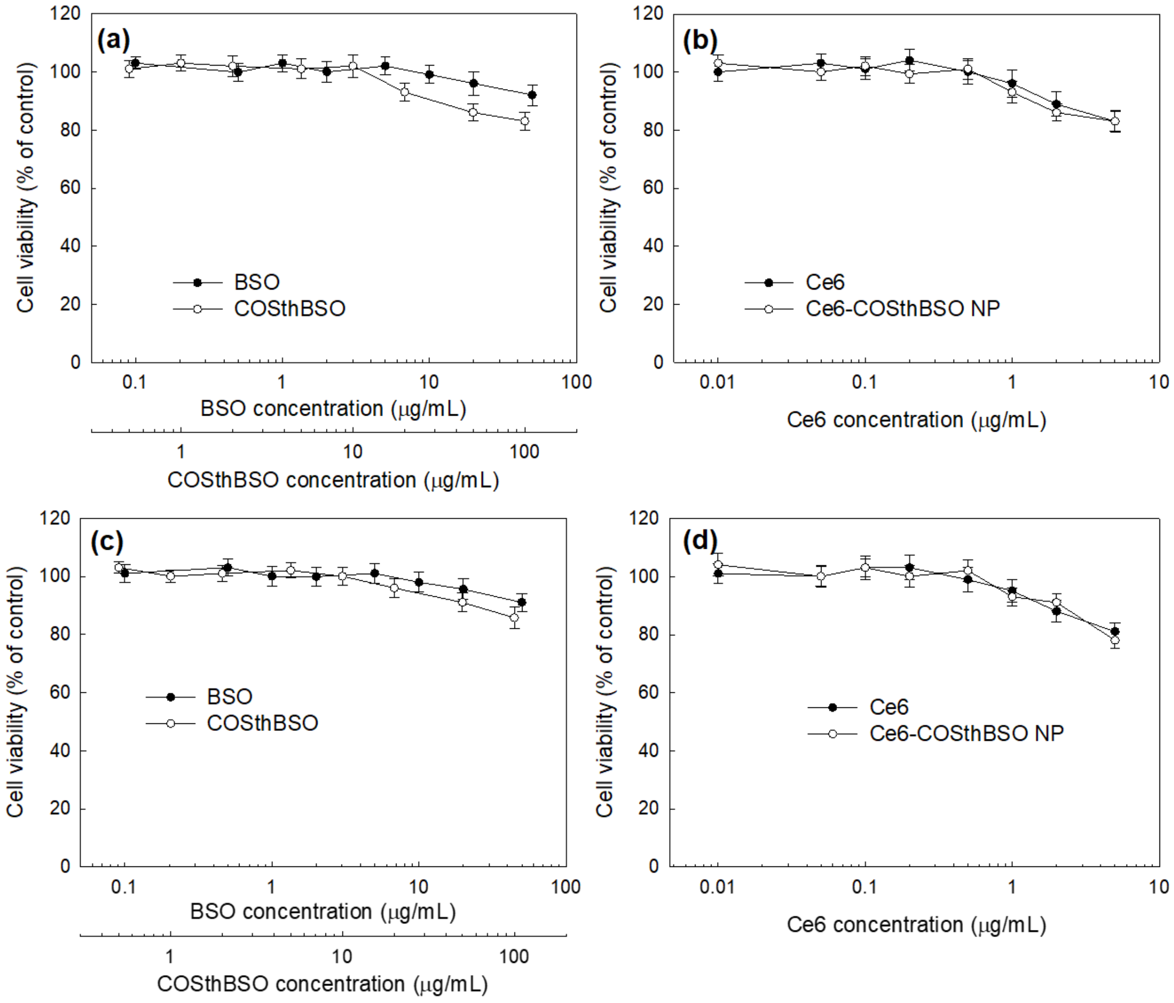

Figure S2. Intrinsic cytotoxicity of BSO, COSthBSO, Ce6 and Ce6-incorporated COSthBSO nanophotosensitizers (COSthBSO-Ce6 NP). CCD986Sk cells (a) and (b); RAW264.7 (c) and (d). BSO and COSthBSO conjugates, (a) and (c); Ce6 and Ce6-COSthBSO NP, (b) and (d).
